# Circulating microRNAs in Response to Exercise Training in Healthy Adults

**DOI:** 10.3389/fgene.2020.00256

**Published:** 2020-03-18

**Authors:** Qiulian Zhou, Chao Shi, Yicheng Lv, Chenglin Zhao, Zheng Jiao, Tianhui Wang

**Affiliations:** ^1^Shanghai Applied Radiation Institute, School of Environmental and Chemical Engineering, Shanghai University, Shanghai, China; ^2^Cardiac Regeneration and Ageing Lab, Institute of Cardiovascular Sciences, School of Life Sciences, Shanghai University, Shanghai, China

**Keywords:** circulating miRNAs, cardiovascular adaptation, acute exercise, cardiopulmonary exercise testing, healthy adults

## Abstract

Circulating microRNAs (miRNAs, miRs) have great potential as cardiac biomarkers and they are also being explored for their roles in intercellular communication and gene expression regulation. The analysis of circulating miRNAs in response to exercise would provide a deeper understanding of the molecular response to physical activity and valuable information for clinical practice. Here, eight male college students were recruited to participate in cardiopulmonary exercise testing (CPET) and 1 h acute exercise training (AET). Blood samples were collected and serum miRNAs involved in angiogenesis, inflammation and enriched in muscle and/or cardiac tissues were analyzed before and after cardiopulmonary exercise and acute exercise. The miRNAs we detected were miR-1, miR-20a, miR-21, miR-126, miR-133a, miR-133b, miR-146, miR155, miR-208a, miR-208b, miR-210, miR-221, miR-222, miR-328, miR-378, miR-499, and miR-940. We found that serum miR-20a was decreased significantly after CPET and serum miR-21 was increased after AET. In addition, no robust correlation was identified between the changes of these miRNAs and makers of cardiac function and exercise capacity, which indicates a distinct adaptation of these miRNAs to exercise. Future studies are highly needed to define the potential use of these circulating miRNAs as useful biomarkers of exercise training, and disclose the biological function of circulating miRNAs as physiological mediators of exercise-induced cardiovascular adaptation.

## Introduction

Regular physical exercise is a part of healthy lifestyle, which can prevent and reduce the risk of diseases, including metabolic and aging-related diseases and even cancer, and affect mitochondrial metabolism as well as cognitive, cardiovascular and immune functions (Moore et al., 2012; Febbraio, 2017). Furthermore, the specific training protocols have become non-pharmacological remedies to reduce cardiovascular morbidity and mortality due to exercise-induced cardiovascular benefits (Liu et al., 2017; Ribeiro et al., 2017). Exercise training could impact multiple signaling pathways and thus influence different exercise-associated traits including energy metabolism, angiogenesis, inflammation, muscle recovery, and mitochondrial biogenesis (Horak et al., 2018). However, the precise cellular and molecular mechanism for beneficial effects of exercise remains unclear. Nevertheless, non-coding RNAs, and especially microRNAs (miRNAs, miRNAs), constitute a new regulatory component that may play a role in exercise-induced adaptations (Silva et al., 2017; Wang et al., 2018).

miRNAs are a group of endogenous small non-coding RNAs of 18–25 nucleotides in length. miRNAs regulate gene expression at post-transcriptional levels through messenger RNA degradation or translational inhibition in a sequence-specific manner (Bartel, 2004, 2007). miRNAs play a role in the progresses of early embryogenesis and proliferation, differentiation, survival, proliferation, apoptosis, metabolism and energy balance of cell (He and Hannon, 2004; Esquela-Kerscher and Slack, 2006). The expression of miRNAs frequently dysregulated in various human diseases, such as inflammation, cardiovascular disease, Alzheimer’s disease, muscle hypertrophy, lymphomas, leukemias, and cancer (Mohamed et al., 2011; Hruska-Plochan et al., 2015; Grobbelaar and Ford, 2019).

Recently, miRNAs have been found to be present in body fluids such as serum, plasma, urine, saliva and cerebrospinal fluid (Weber et al., 2010). Circulating miRNAs are stable, easily detectable, and may regulate gene expression in target cells and tissues as a novel mode of intercellular communication (Kosaka et al., 2010). At present, increasing evidence has reported circulating miRNAs as potential clinical biomarkers for specific diseases and administration of pharmaceutical agents (Zhang et al., 2017a; Caglar and Cayir, 2019; Kiyosawa et al., 2019; Reddy et al., 2019). The changes of circulating miRNAs induced by exercise have been widely reported in healthy persons and patients, indicating miRNAs may play a role in physiological adaptations to exercise. Profiles of circulating miRNAs are varying under different exercise type, duration and intensity (Xu et al., 2016; de Gonzalo-Calvo et al., 2018; Horak et al., 2018; Li et al., 2018; Ramos et al., 2018; Sapp and Hagberg, 2019). However, less is known about the changes of circulating miRNAs in the adaptation to cardiopulmonary exercise testing (CPET) and acute exercise training (AET).

Here, we inspected how CPET and AET affect specific circulating miRNAs with well-established roles in major adaptive processes in healthy persons. Specially, to further determine the uniqueness of circulating miRNAs changes, we compared the changes of circulating miRNAs to cardiac functional indexes and exercise capacity at baseline. We found that serum miR-20a was decreased significantly after CPET, while serum miR-21 was increased after AET. Moreover, no robust correlation was identified between changes of these miRNAs and makers of cardiac function and exercise capacity, indicating further studies using high-throughput circulating miRNAs screening techniques are highly needed to identify the potential role of circulating miRNAs in exercise adaptation.

## Materials and Methods

### Participants

This study is in line with the recommendations of Ethics Committee. All subjects included in the study are fully aware of the methods and objectives of the study, and signed the informed consent voluntarily according to the Declaration of Helsinki. This study recruited eight male college students from Shanghai University with an average age of 20.75 ± 0.46 as the research subjects. Relevant clinical indicators including aortic root dimension, left ventricular end diastolic diameter and ejection fraction (EF) were tested.

Cardiopulmonary exercise testing and 1 h of AET were received by each subject, and clinical cardiac function indictors were collected when their exercise reached the anaerobic threshold (AT) and peak oxygen uptake. For CPET, using the MasterScreen-CPX system (Jaeger, Germany), after a rest of 3 min and an unloaded exercise of 3 min, the workload was increased from 0 J/s for 1 min and increased by 2 J/s ever 6s. All participants continued pedaling until they reached the peak oxygen uptake. For acute exercise, these participants were exercised at Sweden Monark bicycle for 60 min at 70% of peak oxygen uptake. The ECG exercise test of all subjects was negative, and their exercise tolerance and ventilation function were normal.

### The Extraction of Serum

A total of 5 ml of whole blood was collected before and immediately after cardiopulmonary exercise and acute exercise with procoagulant tubes. After gentle mixing, blood samples were left at room temperature within 1 h to allow coagulation to occur. Centrifuge at 3000 rpm for 15 min at 4°C, the supernatant clear liquid was taken and stored in −80°C refrigerator.

### The Extraction of Serum Total RNA

The mirVana^TM^PARIS^TM^ (Ambion, United States) was used to homogenize the samples through special cell lysis buffer to isolate total RNA from the serum samples. Take 200 μl of each serum sample and add exogenous cel-miR-39 (Ribobio Guangzhou) as a reference to ensure a final concentration of 50 pmol/L. Cel-miR-39 was used as control in the subsequent real-time quantitative PCR.

### Determination of Circulating miRNAs Levels

The miRNA primers involved in the experiment are all purchased from Ribobio. The iScript cDNA synthesis kit (Bio-Rad, United States) was used for reverse transcription of miRNAs. The reaction system of cDNA synthesis is 10 μl, and the transcripts were stored at −20°C. SYBR Green (Bio-Rad, United States) fluorescent dye was used for quantitative PCR amplification. The reaction system was 10 μl. The experiment was performed using the Roche LightCycler^®^ 480 Real-Time PCR system. Data analysis was calculated by using the formula 2^(–ΔΔCt)^.

### Statistical Analysis

All data were analyzed by using SPSS.20, and each group of data was expressed as mean ± standard error of mean (SEM). The results before and after exercise were compared by paired sample *T*-test. Correlation analyses were analyzed using the Spearman’s or Pearson’s method as appropriate for data distribution. Statistical significance is defined as P-values < 0.05. Data plots were made by GraphPad Prism.

## Results

### Subject Characteristics Before and After Acute Exercise Training

Eight male college students with an average age of 20.75 ± 0.46 years were enrolled, and the clinical characteristic for them at the baseline were shown in Table 1. Their average height, weight and the BMI are 176.06 ± 1.61 cm, 69.31 ± 1.77 kg, and 22.34 ± 0.38 kg/m^2^, respectively. In the basic state, their average heart rate was 77.00 ± 2.47 beats/min, the systolic blood pressure was 106.50 ± 5.19 mmHg, and the diastolic blood pressure was 72.25 ± 3.21 mmHg. Table 2 lists the detailed general echocardiographic indexes. The average EF was 65.13 ± 1.19%. We analyzed the cardiopulmonary function indexes when their exercise reached the AT and peak oxygen uptake (Table 3). The average heart rate was 121 ± 5.24 and 175.25 ± 3.41 beats/min when their exercise reached the AT and peak oxygen uptake (Peak). Cardiopulmonary exercise tests revealed that the AT VO_2_ and peak VO_2_ were not changed.

**TABLE 1:** Clinical characteristic of participants.

Clinical parameters	Mean ± SEM
Age (year)	20.750.46
Height (cm)	176.061.61
Body mass (kg)	69.311.77
BMI (kg/m^2^)	22.340.38
Heart rate (beats/min)	77.002.47
Systolic blood pressure (mmHg)	106.505.19
Diastolic blood pressure (mmHg)	72.253.21
	

**TABLE 2:** General echocardiographic indexes of participants.

Clinical parameters	Mean ± SEM
Aortic root dimension (mm)	28.751.14
Left ventricular end diastolic diameter (mm)	49.881.02
Left ventricular end systolic diameter (mm)	32.380.92
End-diastolic volume	117.56.00
End-systolic volume	41.252.60
Left atrial dimension (mm)	30.001.05
Interventricular septal thickness (mm)	8.130.21
Left ventricular posterior wall thickness (mm)	8.500.31
Ejection fraction (EF %)	65.131.19
Fractional shortening (FS %)	35.750.98

**TABLE 3:** Cardiopulmonary function indexes in AT and peak.

Clinical parameters	Mean ± SEM	
	
	AT	Peak
Heart rate (beats/min)	1215.24	175.253.41
Systolic blood pressure (mmHg)	1388.54	172.58.03
Diastolic blood pressure (mmHg)	73.132.92	812.10
VO_2_ (ml/min/kg)	18.451.33	31.060.98
Work load (watts)	97.2510.62	169.756.20
METs	5.270.38	8.880.28
70% VO_2_ max (ml/min/kg)	21.74 ± 0.68	
70%VO_2_max work load (watts)	105.5 ± 3.55	

### Cardiopulmonary Exercise Testing Decreased Circulating miR-20a, While Acute Exercise Training Increased Circulating miR-21

**FIGURE 1:**
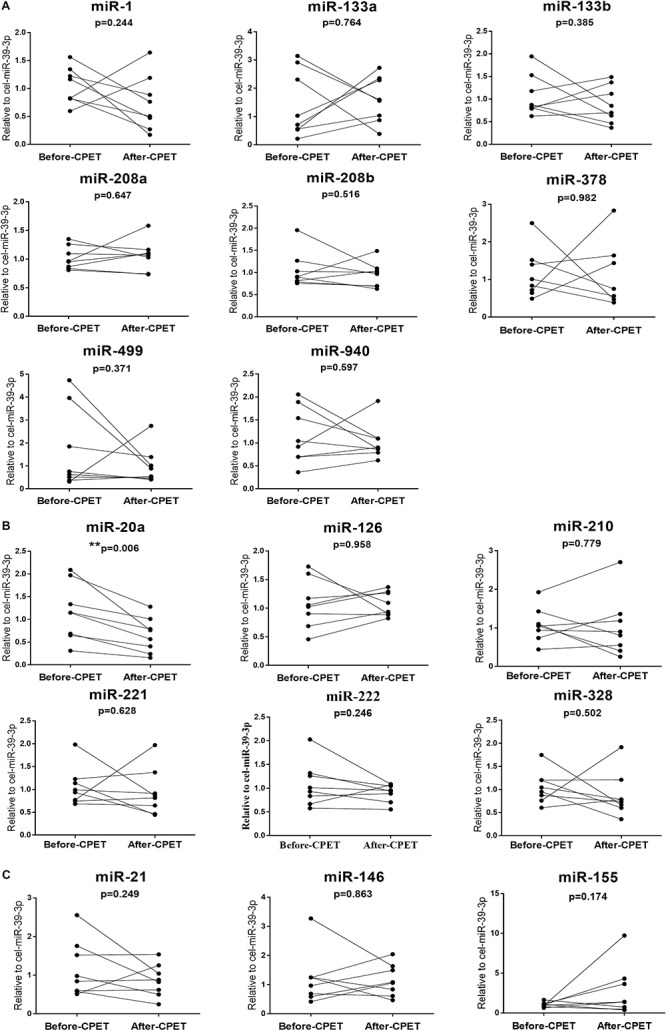
Distinct regulatory profiles of selected circulating miRNAs before and after CPET. **(A)** Serum levels of cardiac or muscle-specific/enriched miRNAs before and after CPET. **(B)** Serum levels of angiogenesis-related miRNAs before and after CPET. **(C)** Serum levels of inflammation-related miRNAs before and after CPET. **P < 0.01; *n* = 8.

**FIGURE 2:**
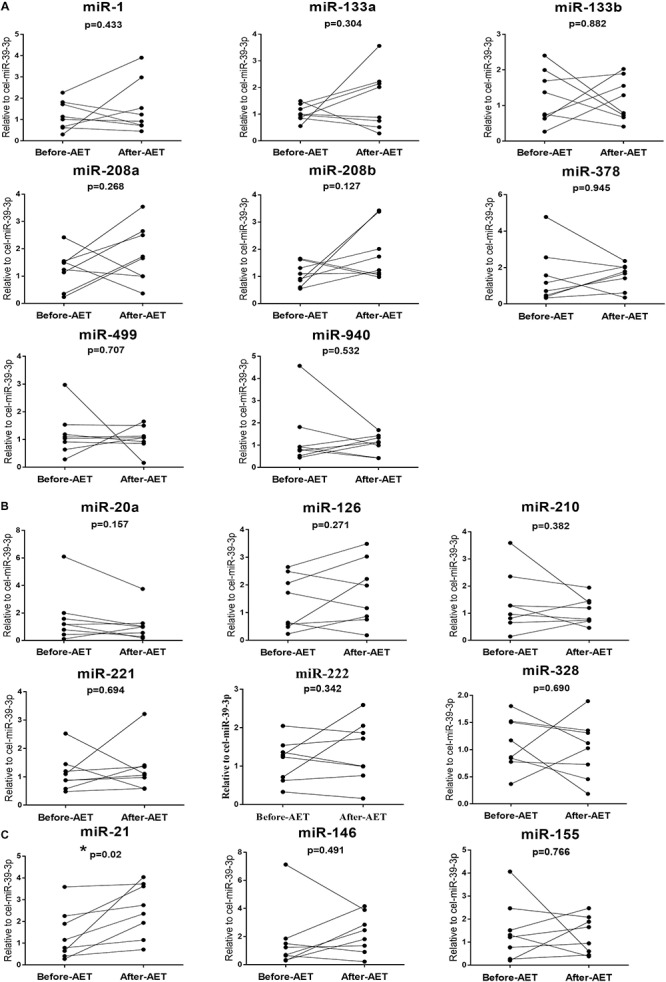
Distinct regulatory profiles of selected circulating miRNAs before and after AET. **(A)** Serum levels of cardiac or muscle-specific/enriched miRNAs before and after AET. **(B)** Serum levels of angiogenesis-related miRNAs before and after AET. **(C)** Serum levels of inflammation-related miRNAs before and after AET. *P < 0.05; *n* = 8.

We determined the expression of cardiac or muscle-specific/enriched miRNAs (miR-1, miR-133a, miR-133b, miR-208a, miR-208b, miR-378, miR-499, miR-940), angiogenesis-related miRNAs (miR-20a, miR-126, miR-210, miR-221, miR-222, miR-328), and inflammation-related miRNAs (miR-21, miR-146, miR155). The results showed that serum miR-20a was decreased significantly after CPET (Figure 1); while serum miR-21 was increased after AET (Figure 2). In contrast, miR-1, miR-133a, miR-133b, miR-208a, miR-208b, miR-378, miR-499, miR-940, miR-126, miR-210, miR-221, miR-222, miR-328, miR-146, and miR-155 were not changed following CPET or AET.

### Correlations Between the Changes of miR-20a Following Cardiopulmonary Exercise Testing and Cardiac Function, Exercise Capacity at Baseline

**FIGURE 3:**
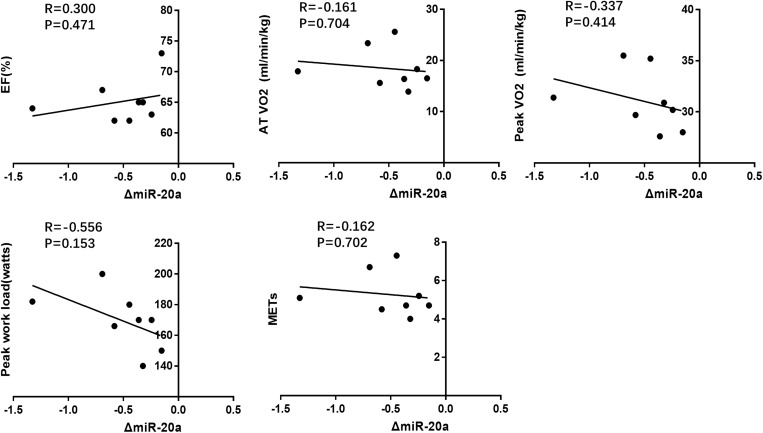
Correlation analysis between the changes of miR-20a following CPET and cardiac function(EF%), exercise capacity (AT VO_2_, peak VO_2_, peak work load, METs) at baseline.

Here, we correlated the decrease of miR-20a after CPET with the cardiac function and exercise capacity at baseline, however, no robust correlations were found (Figure 3 and Table 4). We also failed to find robust correlations between miR-20a and cardiopulmonary function indexes before and after CPET (Table 4).

**TABLE 4:** Correlation analysis between miRNA changes following CPET and cardiopulmonary function indexes.

miR-20a	EF		AT VO_2_		Peak VO_2_		Peak work load		METs	
					
	R	P	R	P	R	P	R	P	R	P
Before CPET	–0.229	NS	0.062	NS	0.386	NS	0.517	NS	0.07	NS
After CPET	–0.085	NS	–0.053	NS	0.305	NS	0.306	NS	–0.042	NS
Δ	0.3	NS	–0.161	NS	–0.337	NS	–0.556	NS	–0.162	NS

### Correlations Between the Changes of miR-21 Following Acute Exercise Training and Cardiac Function, Exercise Capacity at Baseline

**FIGURE 4:**
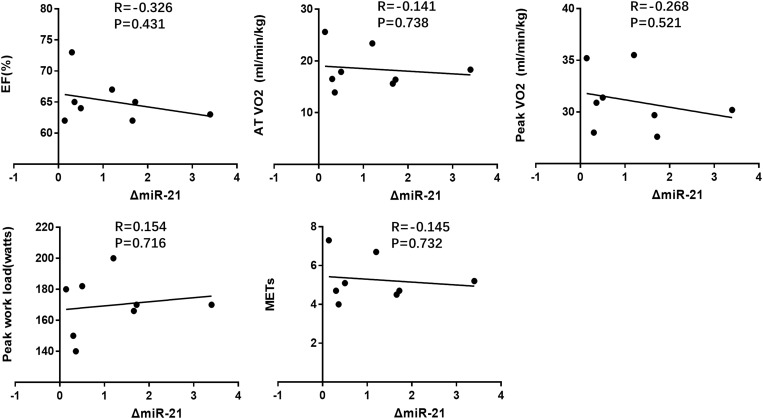
Correlation analysis between the changes of miR-21 following AET and cardiac function (EF%), exercise capacity (AT VO_2_, peak VO_2_, peak work load, METs) at baseline.

We also correlated the increase of miR-21 after AET with the cardiac function and exercise capacity at baseline, however, no robust correlations were found (Figure 4 and Table 5). We also failed to report robust correlations between miR-21 and cardiopulmonary function indexes before and after AET (Table 5).

**TABLE 5:** Correlation analysis between miRNA changes following AET and cardiopulmonary function indexes.

miR-21	EF		AT VO_2_		Peak VO_2_		Peak work load		METs	
					
	R	P	R	P	R	P	R	P	R	P
Before AET	–0.382	NS	0.658	NS	0.506	NS	0.452	NS	0.656	NS
After AET	–0.645	NS	0.482	NS	0.228	NS	0.556	NS	0.477	NS
Δ	–0.326	NS	–0.141	NS	–0.268	NS	0.154	NS	–0.145	NS

## Discussion

The purpose of this study was to investigate how specific circulating miRNAs were regulated by CPET and AET in healthy adults. Whether there are differences among exercise adaptation in athletes, healthy adults, and the patients of specific diseases have been previously published (Xu et al., 2016; Silva et al., 2017; Li et al., 2018). In previous studies, their participants were either chronic heart failure patients or basketball athletes, which is different from healthy college students in this study. The chronic heart failure patients were subjected to a symptom-limited incremental cardiopulmonary exercise test on a bicycle ergometer using a standardized exercise protocol of revised Ramp10 programs. Basketball athletes were subjected to cardiopulmonary exercise and an amateur basketball season for 3 months. In our cohort, these subjects were subjected to cardiopulmonary exercise and acute exercise for 1h, which is different from those in chronic heart failure patients or basketball athletes. Here we reported that serum miR-20a decreased in response to CPET, while serum miR-21 increased in response to AET. We also showed that no robust correlations was identified between the changes of miR-21 and miR-20a induced by exercise to makers of cardiac function and exercise capacity, which suggested that these two miRNAs were distinct biomarkers and also further studies using high-throughput circulating miRNAs screening techniques are highly needed.

A few studies have reported changes in circulating miRNAs after acute and chronic exercise in healthy persons or athletes. Specifically, circulating miR-1, miR-133a, miR-133b, miR-139, miR-143, miR-181b, miR-206, miR-208b, miR-214, miR-223, miR-330, miR-338, miR-485, miR-509, miR-517a, miR-518f, miR-520f, miR-522, miR-553, and miR-888 were found increased, while miR-30b, miR-106a, miR-146a, miR-151, miR-221, miR-652 were decreased after an acute exercise (Banzet et al., 2013; Nielsen et al., 2014; Cui et al., 2016; Silva et al., 2017). Circulating miR-20a, miR-103, miR-107, miR-126, miR-376a were increased, whilemiR-16, miR-21, miR-25, miR-27a, miR-28, miR-148a, miR-185, miR-342, and miR-766 were decreased after a chronic exercise (Baggish et al., 2011, 2014; Nielsen et al., 2014; Zhang et al., 2017b). Circulating miR-21, miR-146a, miR-221, miR-222 were increased in response to both acute and chronic exercise (Baggish et al., 2011). Here we reported that serum miR-20a decreased after CPET and serum miR-21 increased after AET, while other miRNAs measured in this study did not change, suggesting that exercise participants, type, duration and intensity might affect levels of circulating miRNAs. Besides, the sample processing, total RNA isolation method, and miRNAs quantification platforms may also affect levels of circulating miRNAs. Interestingly, most of cardiac or muscle-specific/enriched miRNAs have not changed in this study, there must be different mechanisms of circulating miRNAs response to exercise rather than the general response to tissue damage.

The miRNAs measured in this study have potential biological relevance in exercise, including inflammation, angiogenesis and ischemic adaptation. MiR-21 is abundantly expressed in almost all tissues and involved in many cardiovascular diseases, such as atherosclerosis, pulmonary hypertension, heart failure and myocardial ischemia (Cengiz et al., 2015; Zhao and Zhou, 2017; Pan et al., 2018; Ding et al., 2019; Kan et al., 2019; Sun et al., 2019). MiR-21 plays anti-apoptosis roles in endothelial cells and ischemic cardiomyocytes (Li et al., 2018). The role of miR-21 in response to exercise is to indirectly participate in angiogenesis by inducing the expression of hypoxia inducible factor-1(HIF-1α) and VEGF, regulating apoptosis, increasing the activity of endothelial nitric oxide synthase and mediating anti-inflammatory response in macrophages (Sheedy, 2015; Horak et al., 2018). In this study, we found that serum miR-21 increased in response to AET. We speculated that miR-21 may functions as promoting angiogenesis, anti-inflammatory, and anti-apoptosis effects.

Different from the results of this study, the expression of miR-20a was reported up-regulated in response to sustained aerobic exercise training but no changes to acute exhaustive exercise in athletes (Baggish et al., 2011).Besides, it has also been reported that miR-20a was up-regulated in response to acute resistance exercise in men, which is indicative of a hypertrophic response within skeletal muscle (Margolis et al., 2017). Thus, the adaptive regulation of circulating miRNAs is influenced by exercise type, intensity, duration and human subjects. MiR-20a is able to target the TNFSF15 gene, preventing the latter to function as an inhibitor of angiogenesis. The production of miR-20a is stimulated by the Akt and Erk signals, which is activated by the angiogenic factor VEGF (Wang et al., 2017). In this study, we found that serum miR-20a decreased in response to CPET. We speculated that the decrease of miR-20a was an acute compensatory reaction. As an initial study, several limitations of the present study should be highlighted. Firstly, a major weakness of this study is that the number of enrolled human subjects should be expanded. Secondly, this study was restricted to healthy young persons. Future study is required to determine whether the changes of circulating miRNAs are applicable to the healthy people of different ages and the patients of specific diseases. Thirdly, this study examined circulating miRNAs in response to CPET and acute exhaustive exercise. The dynamic regulation of circulating miRNAs after sustained aerobic exercise training need to be further studied. Fourthly, it would be interesting to see what genes are also influenced by the changed circulating miRNAs. Finally, quantitative analysis was limited to a subset of related miRNAs, and high-throughput screening is needed to obtain a more complete profile of circulating miRNAs regulation in adaption to exercise training. Although further studies are needed, present results contribute to the knowledge about effects of CPET and AET on the circulating miRNAs profile.

## Conclusion

In conclusion, serum miR-20a decreased in response to CPET, while serum miR-21 increased in response to AET. Future studies are highly needed to define the potential use of these circulating miRNAs as useful biomarkers of exercise training, and disclose the direct biological function of circulating miRNAs in adaptation to different modes of exercise training.

## Data Availability Statement

The raw data supporting the conclusions of this article will be made available by the authors, without undue reservation, to any qualified researcher.

## Ethics Statement

The studies involving human participants were reviewed and approved by the Shanghai University Ethics Committee. The patients/participants provided their written informed consent to participate in this study.

## Author Contributions

TW, ZJ, and QZ designed the study, conducted all the experiments, and drafted the manuscript. CS, YL, and CZ conducted the experiments and analyzed the data.

## Conflict of Interest

The authors declare that the research was conducted in the absence of any commercial or financial relationships that could be construed as a potential conflict of interest.

## References

[B1] Baggish A. L. Hale A. Weiner R. B. Lewis G. D. Systrom D. Wang F. (2011). Dynamic regulation of circulating microRNA during acute exhaustive exercise and sustained aerobic exercise training. *J. Physiol.* 589(Pt 16) 3983–3994. 10.1113/jphysiol.2011.213363 21690193 PMC3179997

[B2] Baggish A. L. Park J. Min P. K. Isaacs S. Parker B. A. Thompson P. D. (2014). Rapid upregulation and clearance of distinct circulating microRNAs after prolonged aerobic exercise. *J. Appl. Physiol.* 116 522–531. 10.1152/japplphysiol.01141.2013 24436293 PMC3949215

[B3] Banzet S. Chennaoui M. Girard O. Racinais S. Drogou C. Chalabi H. (2013). Changes in circulating microRNAs levels with exercise modality. *J. Appl. Physiol.* 115 1237–1244. 10.1152/japplphysiol.00075.2013 23950168

[B4] Bartel D. P. (2004). MicroRNAs: genomics, biogenesis, mechanism, and function. *Cell* 116 281–297. 10.1016/s0092-8674(04)00045-5 14744438

[B5] Bartel D. P. (2007). MicroRNAs: genomics, biogenesis, mechanism, and function (Reprinted from Cell, vol 116, pg 281-297, 2004). *Cell* 131 11–29. 10.1016/s0092-8674(04)00045-5 14744438

[B6] Caglar O. Cayir A. (2019). Total circulating cell-free miRNA in plasma as a predictive biomarker of the thyroid diseases. *J. Cell. Biochem.* 120 9016–9022. 10.1002/jcb.28173 30506793

[B7] Cengiz M. Yavuzer S. Avci B. K. Yuruyen M. Yavuzer H. Dikici S. A. (2015). Circulating miR-21 and eNOS in subclinical atherosclerosis in patients with hypertension. *Clin. Exp. Hypertens.* 37 643–649. 10.3109/10641963.2015.1036064 26114349

[B8] Cui S. F. Wang C. Yin X. Tian D. Lu Q. J. Zhang C. Y. (2016). Similar responses of circulating microRNAs to acute high-intensity interval exercise and vigorous-intensity continuous exercise. *Front. Physiol.* 7:102. 10.3389/fphys.2016.00102 27047388 PMC4796030

[B9] de Gonzalo-Calvo D. Davalos A. Fernandez-Sanjurjo M. Amado-Rodriguez L. Diaz-Coto S. Tomas-Zapico C. (2018). Circulating microRNAs as emerging cardiac biomarkers responsive to acute exercise. *Int. J. Cardiol.* 264 130–136. 10.1016/j.ijcard.2018.02.092 29776561

[B10] Ding F. You T. Hou X. D. Yi K. Liu X. G. Zhang P. (2019). MiR-21 regulates pulmonary hypertension in rats via TGF-beta 1/Smad2 signaling pathway. *Eur. Rev. Med. Pharmacol. Sci.* 23 3984–3992. 10.26355/eurrev_201905_17828 31115027

[B11] Esquela-Kerscher A. Slack F. J. (2006). Oncomirs – microRNAs with a role in cancer. *Nat. Rev. Cancer* 6 259–269. 10.1038/nrc1840 16557279

[B12] Febbraio M. A. (2017). Exercise metabolism in 2016: health benefits of exercise more than meets the eye! *Nat. Rev. Endocrinol.* 13 72–74. 10.1038/nrendo.2016.218 28051119

[B13] Grobbelaar C. Ford A. M. (2019). The role of MicroRNA in paediatric acute lymphoblastic leukaemia: challenges for diagnosis and therapy. *J. Oncol.* 2019:894. 10.1155/2019/8941471 31737072 PMC6815594

[B14] He L. Hannon G. J. (2004). Micrornas: small RNAs with a big role in gene regulation. *Nat. Rev. Genet.* 5 522–531. 10.1038/nrg1379 15211354

[B15] Horak M. Zlamal F. Iliev R. Kucera J. Cacek J. Svobodova L. (2018). Exercise-induced circulating microRNA changes in athletes in various training scenarios. *PLoS One* 13:e0191060. 10.1371/journal.pone.0191060 29338015 PMC5770042

[B16] Hruska-Plochan M. Li B. Kyburz D. Kruetzfeldt J. Landmesser U. Aguzzi A. (2015). New and emerging roles of small RNAs in neurodegeneration, muscle, cardiovascular and inflammatory diseases. *Swiss Med. Wkly.* 145:w14192. 10.4414/smw.2015.14192 26376442

[B17] Kan C. L. Cao J. J. Hou J. Jing X. Y. Zhu Y. J. Zhang J. H. (2019). Correlation of miR-21 and BNP with pregnancy-induced hypertension complicated with heart failure and the diagnostic value. *Exp. Ther. Med.* 17 3129–3135. 10.3892/etm.2019.7286 30936985 PMC6434261

[B18] Kiyosawa N. Watanabe K. Toyama K. Ishizuka H. (2019). Circulating miRNA signature as a potential biomarker for the prediction of analgesic efficacy of hydromorphone. *Int. J. Mol. Sci.* 20:1665. 10.3390/ijms20071665 30987164 PMC6480077

[B19] Kosaka N. Iguchi H. Yoshioka Y. Takeshita F. Matsuki Y. Ochiya T. (2010). Secretory mechanisms and intercellular transfer of microRNAs in living cells. *J. Biol. Chem.* 285 17442–17452. 10.1074/jbc.M110.107821 20353945 PMC2878508

[B20] Li Y. Yao M. Zhou Q. Cheng Y. Che L. Xu J. (2018). Dynamic regulation of circulating microRNAs during acute exercise and long-term exercise training in basketball athletes. *Front. Physiol.* 9:282. 10.3389/fphys.2018.00282 29662456 PMC5890107

[B21] Liu X. Platt C. Rosenzweig A. (2017). The role of microRNAs in the cardiac response to exercise. *Cold Spring Harb. Perspect. Med.* 7:a029850. 10.1101/cshperspect.a029850 28389519 PMC5710094

[B22] Margolis L. M. Lessard S. J. Ezzyat Y. Fielding R. A. Rivas D. A. (2017). Circulating microRNA are predictive of aging and acute adaptive response to resistance exercise in men. *J. Gerontol. A Biol. Sci. Med. Sci.* 72 1319–1326. 10.1093/gerona/glw243 27927764 PMC5861902

[B23] Mohamed J. S. Hajira A. Li Z. Paulin D. Boriek A. M. (2011). Desmin regulates airway smooth muscle hypertrophy through early growth-responsive Protein-1 and microRNA-26a. *J. Biol. Chem.* 286 43394–43404. 10.1074/jbc.M111.235127 21903578 PMC3234798

[B24] Moore S. C. Patel A. V. Matthews C. E. de Gonzalez A. B. Park Y. Katki H. A. (2012). Leisure time physical activity of moderate to vigorous intensity and mortality: a large pooled cohort analysis. *PLoS Med.* 9:e1001335. 10.1371/journal.pmed.1001335 23139642 PMC3491006

[B25] Nielsen S. Akerstrom T. Rinnov A. Yfanti C. Scheele C. Pedersen B. K. (2014). The miRNA plasma signature in response to acute aerobic exercise and endurance training. *PLoS One* 9:e87308. 10.1371/journal.pone.0087308 24586268 PMC3929352

[B26] Pan Y. Q. Li X. W. Li Y. C. Li J. L. Lin J. F. (2018). Effect of miR-21/TLR4/NF-kappa B pathway on myocardial apoptosis in rats with myocardial ischemia-reperfusion. *Eur. Rev. Med. Pharmacol. Sci.* 22 7928–7937. 10.26355/eurrev_201811_16420 30536340

[B27] Ramos A. E. Lo C. Estephan L. E. Tai Y.-Y. Tang Y. Zhao J. (2018). Specific circulating microRNAs display dose-dependent responses to variable intensity and duration of endurance exercise. *Am. J. Physiol. Heart Circ. Physiol.* 315 H273–H283. 10.1152/ajpheart.00741.2017 29600898 PMC6139619

[B28] Reddy L. L. Shah S. A. V. Ponde C. K. Rajani R. M. Ashavaid T. F. (2019). Circulating miRNA-33: a potential biomarker in patients with coronary artery disease. *Biomarkers* 24 36–42. 10.1080/1354750x.2018.1501760 30022694

[B29] Ribeiro P. A. B. Boidin M. Juneau M. Nigam A. Gayda M. (2017). High-intensity interval training in patients with coronary heart disease: prescription models and perspectives. *Ann. Phys. Rehabil. Med.* 60 50–57. 10.1016/j.rehab.2016.04.004 27346629

[B30] Sapp R. M. Hagberg J. M. (2019). Circulating microRNAs: advances in exercise physiology. *Curr. Opin. Physiol.* 10 1–9. 10.1016/j.cophys.2019.03.004

[B31] Sheedy F. J. (2015). Turning 21: induction of miR-21 as a key swith in the inflammatory response. *Front. Immunol.* 6:19. 10.3389/fimmu.2015.00019 25688245 PMC4310327

[B32] Silva G. J. J. Bye A. el Azzouzi H. Wisloff U. (2017). MicroRNAs as important regulators of exercise adaptation. *Prog. Cardiovasc. Dis.* 60 130–151. 10.1016/j.pcad.2017.06.003 28666746

[B33] Sun P. Tang L. N. Li G. Z. Xu Z. L. Xu Q. H. Wang M. (2019). Effects of MiR-21 on the proliferation and migration of vascular smooth muscle cells in rats with atherosclerosis via the Akt/ERK signaling pathway. *Eur. Rev. Med. Pharmacol. Sci.* 23 2216–2222. 10.26355/eurrev_201903_17269 30915769

[B34] Wang D. W. Wang Y. Ma J. Wang W. P. Sun B. B. Zheng T. F. (2017). MicroRNA-20a participates in the aerobic exercise-based prevention of coronary artery disease by targeting PTEN. *Biomed. Pharmacother.* 95 756–763. 10.1016/j.biopha.2017.08.086 28888922

[B35] Wang L. J. Lv Y. C. Li G. P. Xiao J. J. (2018). MicroRNAs in heart and circulation during physical exercise. *J. Sport Health Sci.* 7 433–441. 10.1016/j.jshs.2018.09.008 30450252 PMC6226555

[B36] Weber J. A. Baxter D. H. Zhang S. Huang D. Y. Huang K. H. Lee M. J. (2010). The microRNA spectrum in 12 body fluids. *Clin. Chem.* 56 1733–1741. 10.1373/clinchem.2010.147405 20847327 PMC4846276

[B37] Xu T. Zhou Q. Che L. Das S. Wang L. Jiang J. (2016). Circulating miR-21, miR-378, and miR-940 increase in response to an acute exhaustive exercise in chronic heart failure patients. *Oncotarget* 7 12414–12425. 10.18632/oncotarget.6966 26799589 PMC4914295

[B38] Zhang J. Xing Q. Zhou X. Li J. Li Y. Zhang L. (2017a). Circulating miRNA-21 is a promising biomarker for heart failure. *Mol. Med. Rep.* 16 7766–7774. 10.3892/mmr.2017.7575 28944900

[B39] Zhang T. Brinkley T. E. Liu K. Q. Feng X. Marsh A. P. Kritchevsky S. (2017b). Circulating MiRNAs as biomarkers of gait speed responses to aerobic exercise training in obese older adults. *Aging (Albany N. Y.)* 9 900–913. 10.18632/aging.101199 28301325 PMC5391238

[B40] Zhao Z. Y. Zhou Y. M. (2017). Circulating miR-21 and miR-423-5p as biomarkers for heart failure in heart valve disease patients. *Int. J. Clin. Exp. Pathol.* 10 5703–5711.

